# Influence of Heat Treatments on Carotenoid Content of Cherry Tomatoes

**DOI:** 10.3390/foods2030352

**Published:** 2013-07-31

**Authors:** Laura D’Evoli, Ginevra Lombardi-Boccia, Massimo Lucarini

**Affiliations:** CRA-NUT, Agricultural Research Council-Center for Food and Nutrition, Via Ardeatina 546, Rome 00178, Italy; E-Mails: lombardiboccia@inran.it (G.L.-B.); lucarini@inran.it (M.L.)

**Keywords:** lycopene, home-processed, thermal treatments, geometric isomers

## Abstract

Tomatoes and tomato products are rich sources of carotenoids—principally lycopene, followed by β-carotene and lutein. The aim of this work was to study the effect of heat treatment on carotenoid content in cherry tomatoes. Raw and canned products were sampled and analysed; furthermore whole, skin and pulp fractions of cherry tomatoes were analysed when raw and home-processed, in order to better understand heat treatment effects. Lycopene content in canned tomatoes was two-fold higher than in raw tomatoes (11.60 mg/100 g *versus* 5.12 mg/100 g). Lutein and β-carotene were respectively 0.15 mg/100 g and 0.75 mg/100 g in canned tomatoes *versus* 0.11 mg/100 g and 1.00 mg/100 g in raw tomatoes. For home-processed tomatoes, β-carotene and lutein showed a content decrease in all thermally treated products. This decrease was more evident for β-carotene in the skin fraction (−17%), while for lutein it was greater in the pulp fraction (−25%). Lycopene presented a different pattern: after heat treatment its concentration increased both in the whole and in pulp fractions, while in the skin fraction it decreased dramatically (−36%). The analysis of the isomers formed during the thermal treatment suggests that lycopene is rather stable inside the tomato matrix.

## 1. Introduction

Carotenoids are pigments ubiquitous in nature and responsible for the bright yellow to dark red colour of vegetal products. Numerous epidemiological studies have demonstrated a relationship between carotenoid intake and reduction in risk of developing degenerative diseases [1,2,3,4,5,6,7,8].

Among vegetables, tomatoes and tomato products are rich sources of carotenoids [9,10]. Lycopene, the most abundant pigment (60%–64%), is responsible for red colouration [11]. The antioxidant capacity, together with provitaminic properties typical of other tomato carotenoids [12,13], have drawn attention towards this widely consumed fruit over many years. A number of epidemiological studies have shown that a large consumption of lycopene-rich food is inversely correlated with the risk of developing prostate cancer, while intake of fruit and vegetables is not related with these types of tumours [14,15,16]. Studies on bioavailability provides evidence that industrial tomato products are a better source of carotenoids than raw ones [17,18,19,20]: in particular lycopene extractability significantly increases after thermal pasteurization and mechanic homogenization treatments due to the breaking of protein complexes where the pigment is associated inside the vegetable matrix [21,22,23,24].

In a study by van het Hof *et al.* [25] the different effects of homogenization and heating on carotenoids bioavailability from tomatoes have been evaluated: both treatments increased bioavailability and high-pressure homogenization had a greater impact than normal pressure homogenization, since it breaks extra cell membranes. Further studies on fruits and vegetables also confirmed that bioaccessibility of carotenoids is enhanced by thermal and mechanical processing [26,27,28,29]. 

Thermal treatment also results in isomerization of *E* to *Z* forms [30]: while β-carotene isomerized rapidly, lycopene showed a greater molecular stability inside the biological matrix [31,32,33]. Anguelova and Warthesen [34] studied lycopene stability in tomato freeze-dried products during the various phases of storage, observing an increase by 14%–18% of *Z* forms with respect to total all *E* form. Moreover, individual lycopene and β-carotene stability was found to be strongly related to the processing temperature in tomatoes submitted to hot air drying [35].

Few studies aimed at evaluating the effects of technological treatments on the fruit texture have been carried out on cherry tomatoes [36,37,38], a widely available variety of tomato which is currently consumed both as fresh and as industrially canned product; not much data are available about carotenoids content and their antioxidant capacity in cherry tomato [39,40,41,42,43]. It is known that lycopene is mostly located in tomato skin. In addition, the only data on carotenoid distribution in tomato fractions are limited to lycopene and are not related to the cherry tomatoes variety [44]. Although tomatoes contain many carotenoids, such as γ-carotene, neurosporene, phytofluene and phytoene [45], the present research focuses on lutein, lycopene and β-carotene distribution in raw and canned cherry tomatoes, studying the effect of thermal treatment on the stability of the analysed carotenoids in the products as a whole, skin and pulp fractions.

## 2. Experimental

### 2.1. Samples Preparation: Raw, Home-Processed and Canned Tomatoes

Raw cherry tomatoes grown in Pachino (Sicily), the main area of production in Italy, and collected in the month of July, were pooled and divided into three portions of about 1 kg each: one portion was sent directly to analysis (raw cherry); the second portion was heated in laboratory simulating the industrial canning procedure [37]: aliquots of samples were distributed in 250 mL glass jars provided with hermetic seal and immersed in boiling water for 20 min (home-processed cherry). The third portion was manually separated into pulp and skin fractions after blanching in boiling water for 1 min. The resulted fractions were analysed both as raw (raw pulp; raw skin) and as home-processed following the same procedure as applied for the whole sample (home-processed pulp; home-processed skin). Canned industrial products of the most representative brands were purchased from various markets in Rome (Italy). It was not possible to analyse canned samples coming from the same batch of the fruit destined to processing because these items were manufactured only on an industrial scale. Six cans (250 g), containing whole cherry tomatoes and tomato juice each, were refrigerated at 4 °C, homogenized by a blender (Tecator 1094 Homogenizer, Tecator, Hoganas, Sweden) and frozen at −30 °C until analysis (canned cherry); the addition of tomato juice is a common manufacturing practice in Italy, carried out in order to produce samples with more thickness, colour and taste.

All pooled samples were homogenized by blending and frozen at −30 °C until analysis. Moisture content was determined for each sample by means of a triple analysis.

### 2.2. Reagents

HPLC solvents tetrahydrofuran (THF), methylene chloride (CH_2_Cl_2_), acetonitrile (CH_3_CN), methanol (CH_3_OH), sodium chloride (NaCl) and anhydrous sodium sulfate (Na_2_SO_4_) were purchased from J.T. Baker (Deventer, Holland). THF was stabilized with 2,6-di-*tert*-butyl-*p*-cresol (BHT) 0.01% (Fluka Chemicals, Buchs, Switzerland), calcium carbonate was purchased from Carlo Erba (Milan, Italy).

Chromatographic standards for lutein, lycopene, β-carotene (all *E* form) and internal standard (ISTD) β-apo-8′-carotenal were purchased from Sigma-Aldrich (St. Louis, MO, USA).

### 2.3. Extraction and HPLC Analysis of Carotenoids

Carotenoid extraction was carried out according to the method described by Tonucci *et al.* [46]. Extraction was performed at 0 °C using amber glassware, in order to avoid carotenoids photoxidation. About 50 g sample were homogenised in a beaker by Ultraturrax (IKA Labortechnik, model T-25, Staufen, Germany) with 150 mL of THF and calcium carbonate. The extract was filtered and the solid residue was extracted with 150 mL of THF until complete loss of colour. Extracted fractions were combined and concentrated in a rotary vacuum at 25 °C until reaching one third of the starting volume; the reduced volume was then transferred in a separator funnel with 75 mL of CH_2_Cl_2 _and 45 mL of salted water (10% NaCl); this procedure was repeated two or more times to allow complete carotenoid separation. The organic layer was then filtered on anhydrous Na_2_SO_4 _and concentrated in a rotary vacuum at 25 °C to a final volume of 15 mL. Complete drying was avoided, since lycopene is partially degraded in these conditions. The solution was then filled to a final volume of 50 mL with mobile phase: 20 μL of such volume were injected into the column, after filtering on a 0.45 µm PTFE filter. The chromatographic analysis was performed on a HPLC equipment provided with a quaternary pump (Waters, model 600, Milford, MA, USA) coupled with a photodiode array detector (PAD) (Waters, model 996, Milford, MA, USA). Carotenoid separation was carried out on reversed phase (column C_18_ Inertsil ODS, 250 × 4.6 mm, GL Sciences Inc., Tokyo, Japan) and the elution was obtained using a mobile phase composed by a mixture of CH_3_CN/CH_2_Cl_2_/CH_3_OH (70/20/10), degassed for about 10 min with an helium flow (sparge 100%) before entering the column and sparging 30% during the run; the elution flow rate was 1 mL/min. 

### 2.4. Standard Solutions

Lycopene and β-carotene were dissolved in hexane, lutein was dissolved in ethanol and internal standard β-apo-8′-carotenal in petroleum ether. The solutions were titrated by spectrophotometric analysis and concentrations were calculated from their respective extinction coefficients [47]. Calibration curves were constructed by injection in column of various standard dilutions from a stock solution: the range for these dilutions was chosen in order to include the average concentration at which each carotenoid occurred in the sample. Standard recovery resulted in over 94%.

### 2.5. Statistical Analysis

Each sample was extracted three times, and two consecutive column injections for each analysis were performed. All data were statistically elaborated; means and standard deviations were calculated and a Student’s *t* test used for significant differences found between raw and heated samples was applied.

## 3. Results and Discussion

**Figure 1:**
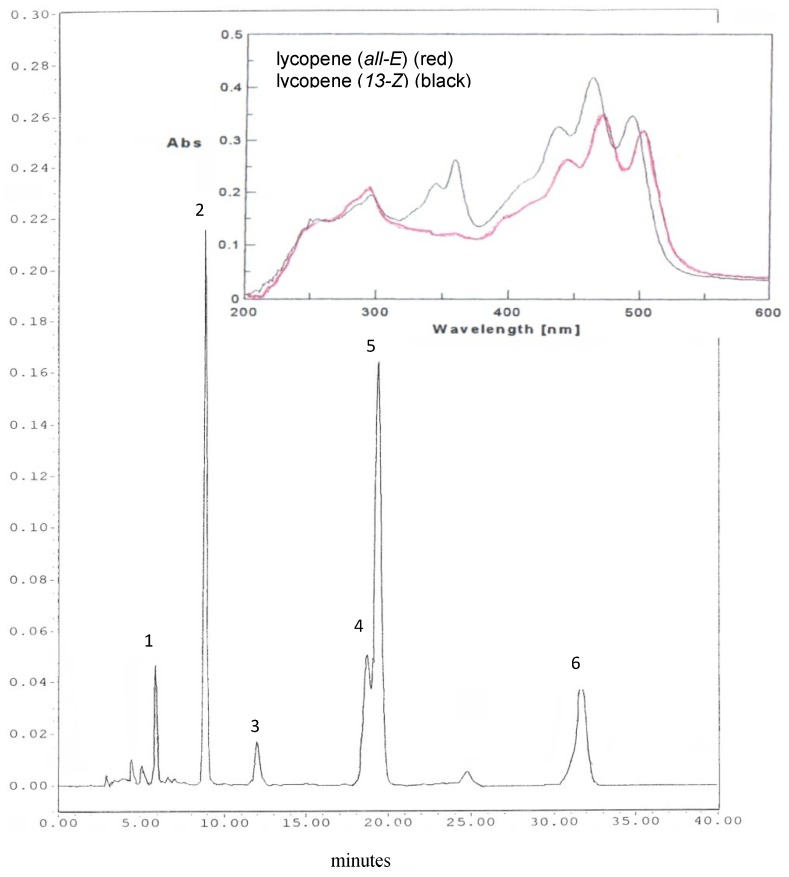
Carotenoid chromatographic separation in raw cherry tomato. Peaks: 1, lutein; 2, β-apo-8′-carotenal (ISTD); 3, unidentified peak (supposed lycopene *Z* isomer); 4, lycopene 13-*Z*; 5, lycopene all *E*; 6, β-carotene.

Carotenoids were eluted in 35 min (Figure 1): lutein, lycopene and β-carotene were identified by comparison of UV-Vis spectra and retention times of correspondent analytical standards, which were previously titrated and injected. The identification of one of the main isomers of lycopene (13-*Z*-lycopene) was made by comparing the UV-Vis spectra with that reported in literature, characterized by an absorption band at about 300 nm along with three characteristic carotenoids bands between 400 and 500 nm [34].

Data from raw and industrially canned samples are reported in Table 1. Lycopene was the most abundant carotenoid, with values ranging from 5.12 mg/100 g fresh weight (f.w.) in the raw sample to 11.60 mg/100 g f.w. in the industrial one. Lutein and β-carotene occurred in lower amounts: lutein content was 0.11 mg/100 g f.w. when raw and 0.15 mg/100 g f.w. in the industrial sample. β-Carotene content varied from 1.00 mg/100 g f.w. in the raw sample to 0.75 mg/100 g f.w. in the canned sample. Leonardi *et al.* [39] studied raw cherry tomatoes from two Sicilian areas (Ragusa and Siracusa), grown in a greenhouse and irrigated with water at different salt concentration for which they found higher values for lycopene (7.20 mg/100 g f.w. and 10.80 mg/100 g f.w.) and similar values for β-carotene (0.92 mg/100 g f.w. and 1.05 mg/100g f.w.).

**Table 1:** Carotenoid contents in raw and canned cherry tomatoes (mg/100g f.w.), values are mean ± standard deviation of three determinations).

Samples	Moisture	Lutein	Lycopene	β-Carotene
	g/100 g	mg/100 g f.w.	mg/100 g f.w.	mg/100 g f.w.
Raw	93.70 ± 0.10	0.11 ± 0.00	5.12 ± 0.35	1.00 ± 0.05
Canned	92.50 ± 0.16	0.15 ± 0.01	11.60 ± 0.18	0.75 ± 0.01

Raffo *et al.* [40] analysed carotenoid content of cherry tomatoes at five different ripening stages; values found for lycopene and β-carotene in our work were comparable to those for tomatoes at an intermediate ripening stage. Other data about carotenoids in cherry tomato were reported by Holland *et al.* [48], where only β-carotene content (0.46 mg/100 g f.w.) in raw sample was indicated.

In a study on composition of eight tomato varieties, different values were provided by Zanfini *et al.* [43] who reported a much higher lycopene content of 11.27 mg/100 g f.w. and a much lower lutein content of 0.02 mg/100 g f.w. in raw cherry tomatoes (cv. Naomi).

A great variation for lycopene and β-carotene content in cherry tomatoes from different origin was reported by Adalid *et al.* [49], lycopene ranging from 0.70 mg/100 g f.w. to 12.00 mg/100 g f.w., and β-carotene from 0.40 mg/100 g f.w. to 1.45 mg/100 g f.w. 

Although a direct comparison is not possible between raw and canned samples, as they did not come from the same batch and slight differences in carotenoid content could already be present in the raw cherry tomatoes destined to canning, it is evident that lycopene concentration was much higher in the industrial canned samples than the raw samples. This may be partially due to the technological treatments of pasteurization and homogenization of canned products, which can improve the extractability of the pigment from the vegetable matrix, although it may be also ascribed to the addition of tomato juice deriving from high ripening stage tomatoes with a great content of lycopene.

In order to study the effect of technological treatments of pasteurization and homogenization, samples of whole cherry tomato and of pulp and skin fractions were processed in laboratory, under industrial-like conditions. Table 2 compares carotenoid values in raw and home-processed fractions. The distribution of carotenoid content in the analysed fractions was not homogeneous; specifically lycopene occurred in skin fractions with concentrations about four-fold higher than in pulp fraction. This result is consistent with data reported by Al-Wandawi *et al.* [50] and those of Sharma and Le Maguer [44], who found a lycopene content about four-fold higher in skins than in pulp in various tomato cultivars. Also β-carotene content resulted higher in skin compared to pulp fraction, but at a lower extent than lycopene. 

**Table 2:** Carotenoid contents in raw and processed cherry tomato fractions. Values are mean ± standard deviation of three determinations. Level of significance raw *versus* home-processed: *** *p* < 0.001; ** *p* < 0.01; * *p* < 0.05.

	Carotenoid (mg/100 g f.w.)					
	Whole		Pulp		Skin	
	Raw	Home-processed	Raw	Home-processed	Raw	Home-processed
Lutein	0.11 ± 0.00	0.10 ± 0.01	0.12 ± 0.01	0.09 ± 0.01 *	0.09 ± 0.01	0.08 ± 0.00
Lycopene	5.12 ± 0.35	5.58 ± 0.35	4.54 ± 0.11	5.39 ± 0.26 **	19.24 ± 0.76	12.25 ± 1.11 ***
β-Carotene	1.00 ± 0.05	0.93± 0.06	1.07 ± 0.01	1.01 ± 0.01 **	1.38 ± 0.04	1.15 ± 0.04 **

β-Carotene and lutein showed a content decrease in all heated products. For β-carotene the decrement was more evident in skin fraction (−17%), while for lutein was greater in pulp fraction (−25%). Lycopene presented a different pattern: after heat treatment its concentration increased both in whole and in pulp (*p* < 0.01), while decreased dramatically in skin fraction (−36%).

It may be assumed that heating caused both pigment degradation and an extractability increase due to the breaking of protein-carotenoid complexes: in the skin the first effect prevailed because of the thinness of tissue, which allowed easy and fast heat conduction; by contrast, in the pulp the lower temperatures reached allowed the promotion of the breakdown of protein complexes with lycopene, but not its degradation. The majority of lycopene molecules may be not bound to proteins in the skin, but to the portion of insoluble fibre [50], thus resulting more sensitive to thermal treatment effects.

Results from a study by Abushita *et al.* [13] on the effects of technological treatments on tomato carotenoids were found to be consistent with those in the present research: analysis performed in raw and thermally and mechanically treated (removal of seeds and skin) salad tomatoes demonstrated that lycopene extractability increased after heating (+37%), while β-carotene and lutein were partially degraded by heat (−29% and −3%, respectively).

Isomerization phenomenon of carotenoids during heating was also investigated in whole cherry tomatoes. A lycopene *Z* form was already detected in the raw sample—it was hypothesized to be the lycopene 13-*Z* from its UV-Vis spectrum, evidenced by an absorption band in the 300–400 nm zone. Also, a shoulder of β-carotene peak corresponding to the formation of a *Z* isomer could be observed in the heated sample, but it was not possible to separate and quantify it under the adopted chromatographic conditions. Lutein is also prone to isomerization and degradation during heating, however no *Z* isomeric forms were detected at the current analytical conditions.

Lycopene 13-*Z* isomer concentration was expressed by normalizing its area with that of the relative all *E* isomer: a small increase in lycopene *Z* isomer was observed during heating (6%, *p* < 0.05), rising from 23% in raw samples to 29% in home-processed samples. Studies about carotenoids isomerization confirm this result: Nguyen *et al.* [51] found that lutein and β-carotene *Z* isomers increased by 27% and 21%, respectively, after heating at 100 °C for 30 min, while lycopene resulted to be stable to isomerization.

Some lycopene stability was also observed by Knockaert [33] who studied degradation and isomerization degree of lycopene in tomato puree after thermal treatment (pasteurization and sterilization) and high pressure homogenization, finding a different *Z* isoform formation during heat treatment. Nguyen *et al.* [51] correlated the greater stability of lycopene with respect to β-carotene mainly to two hypotheses: the first is of thermodynamic nature and is related to the different chemical structure of the two carotenoids, and therefore correlated to the different activation energies involved in the isomerization process. The second, more probable, is related to the different intracellular localization of the two pigments: lycopene, having a linear chemical structure, is more stable during *E*-*Z* conversion because of molecular aggregation as crystals in the chloroplasts. Heating therefore, does not strongly affect lycopene chemical structure as long as it is in the vegetable matrix.

From a nutritional point of view, the *Z* forms of β-carotene show a lower provitaminic A activity than the all *E* form [52]: β-carotene 9-*Z* and β-carotene 13-*Z* provitaminic activity was respectively 38% and 62% compared to that from all *E* form. Concerning the antioxidant power, the *Z* forms of the analysed carotenoids seem to exert a greater antioxidant activity than the equivalent all *E* form [53]. For instance the *in vitro* antioxidant activity of lycopene 9-*Z* was found to be 30% higher compared to all *E* form [54].

Heating leads to a slight increase in lycopene *Z* forms (<10%) and thus does not account directly for the great difference in *E*-*Z* ratio evidenced in plasma and tissues (>58%) [55]; also lycopene *Z* isomers would seem to be preferentially absorbed at intestinal level compared with all *E* isomer [56,57,58].

## 4. Conclusions

Analyses performed in this study leads to three interesting conclusions with regard to the effect of heating on carotenoids distribution in cherry tomato: partial degradation of all pigments, lycopene and β-carotene isomerization from *E* to *Z* forms and increased lycopene extractability from the vegetable matrix. This allowed the assessment of the stability of the three carotenoids: lycopene was observed to be more stable when bound to proteins inside the vegetable matrix—due to protein denaturation and breakdown of crystal aggregates by heating process, the pigment was more extractable. On the other hand, when lycopene was not protein-bound, it was markedly less stable than the other two carotenoids considered (this phenomenon was evident in the skin fraction). The study conducted on the isomerization process in the whole product confirmed that lycopene was more stable inside the vegetable matrix.

The elevated differences in lycopene content between raw and industrial canned products can be only partially attributed to the heating effect, while the different pedoclimatic conditions [39], the use of overripe tomatoes [59] and the addition of ripe tomato juice were likely to be the most responsible factors.
